# Artificial Intelligence in Orthopaedic Research: A Technical Report on Opportunities and Pitfalls

**DOI:** 10.7759/cureus.104159

**Published:** 2026-02-23

**Authors:** Anirudh Dwajan

**Affiliations:** 1 Orthopaedics, All India Institute of Medical Sciences, Bilaspur, IND

**Keywords:** artificial intelligence, deep learning, diagnostic imaging, gait analysis, generative ai, machine learning, orthopaedic research, predictive modeling, research ethics, surgical planning

## Abstract

Artificial intelligence is transforming the landscape of orthopaedic research, offering tools that enhance data analysis, improve diagnostic workflows, and support personalized patient care. In recent years, AI applications in orthopaedics have expanded significantly, ranging from imaging-based fracture detection and musculoskeletal tumor classification to surgical planning, implant identification, and biomechanical gait analysis. Additionally, AI is being used in research-centric tasks, including outcome prediction modeling, literature screening, and preliminary manuscript drafting.

This technical report presents a narrative technical review synthesizing emerging applications of AI within orthopaedic research based on recent PubMed-indexed studies from the past five years. We explore how machine learning and deep learning algorithms are being developed, validated, and deployed across various research domains. The report highlights the tangible benefits of AI, such as increased efficiency, diagnostic precision, and reproducibility of analysis. However, it also addresses the significant pitfalls, including reliance on limited or biased datasets, lack of model transparency, and unresolved ethical challenges.

Of particular concern is the use of generative AI tools in scientific writing, which, while promising, raises questions about originality, accuracy, and research integrity. Overall, AI is poised to support, not replace, orthopaedic researchers. Successful integration will require robust validation, ethical safeguards, and continued collaboration between data scientists and clinical experts.

## Introduction

Orthopaedic care continues to face several practical challenges, including increasing patient load, variability in diagnostic interpretation among clinicians, and delays in imaging review and treatment planning. These limitations can influence patient outcomes and healthcare efficiency. Advances in computational technologies, particularly AI, are increasingly being explored as potential solutions to support clinical decision-making and streamline workflow processes.

Artificial intelligence is increasingly influencing orthopaedic research and practice, offering powerful tools for data analysis, clinical decision support, and workflow automation. Key applications include machine learning, deep learning, and natural language processing for fracture detection, scoliosis curve classification, implant recognition, and surgical navigation systems [1-4]. Artificial intelligence includes a group of computational techniques designed to recognise patterns and make predictions from large datasets. Machine learning refers to algorithms that improve their performance by learning from data rather than following fixed programming rules. Deep learning is a more advanced form of machine learning that uses layered neural networks to analyse complex, high-dimensional data, particularly in medical imaging.

Convolutional neural networks (CNNs) are a specialised deep learning model particularly suited for analysing visual data such as radiographs and MRI scans. Natural language processing (NLP) enables computers to interpret and generate human language, allowing applications such as automated literature review, clinical documentation analysis, and manuscript drafting support. Artificial intelligence, therefore, represents a collection of related technologies that differ in technical design but share a common goal of improving data interpretation and clinical insight [5,6]. In research contexts, AI has also been applied to outcome prediction, biomechanical analysis, and academic support tasks such as literature screening and automated manuscript generation [1,3].

The core advantage of AI lies in its ability to analyse large volumes of complex data rapidly, detect subtle patterns, and generate predictive insights that may enhance diagnostic accuracy, streamline surgical planning, and optimise research efficiency. For example, CNNs have demonstrated performance comparable to expert radiologists in detecting musculoskeletal injuries, while supervised learning models are being used to forecast postoperative outcomes and hospital resource utilisation [7].

However, integrating AI into orthopaedic research is not without risks. Common challenges include limited training datasets, lack of generalizability, opaque algorithmic processes, and ethical issues surrounding academic misuse and over-reliance on automation. Generative AI tools, while promising for knowledge synthesis, are particularly vulnerable to creating inaccurate or “hallucinated” outputs, requiring strong human oversight.

The expanding body of literature exploring AI in orthopaedics highlights the need for a structured overview of its clinical and research applications. This technical report presents a narrative review of PubMed-indexed studies published between 2020 and 2025 that examine the role of artificial intelligence in orthopaedic practice and research. Relevant articles were identified using search terms including “artificial intelligence,” “machine learning,” “deep learning,” and “orthopaedics.” Studies were selected based on their relevance to key thematic areas, including diagnostic imaging, surgical planning, biomechanics and gait analysis, predictive clinical modelling, and academic research workflows. The objective of this report is to summarise current developments while critically examining methodological limitations, ethical considerations, and future research directions.

## Technical report

Methods

This manuscript represents a narrative technical review of AI applications in orthopaedics. A literature search was performed using the PubMed database to identify relevant articles published between January 2020 and January 2025. Search terms included combinations of “artificial intelligence,” “machine learning,” “deep learning,” “orthopaedics,” “fracture detection,” “surgical planning,” “gait analysis,” and “predictive modelling.”

Articles were selected based on relevance to clinical or research applications of artificial intelligence in orthopaedics. Both clinical studies and technical validation studies were considered. Editorials, opinion pieces, and non-English articles were excluded. Given the narrative scope of this review, formal systematic review methodology and quantitative meta-analysis were not performed. Instead, studies were synthesised thematically to describe current applications, methodological strengths and limitations, and future research directions. Where available, performance metrics such as accuracy, sensitivity, specificity, and area under the curve were extracted from representative studies to illustrate reported outcomes.

Diagnostic imaging applications

Among the various applications of AI in orthopaedics, diagnostic imaging remains the most mature and widely studied. The AI tools have demonstrated strong performance in detecting musculoskeletal conditions such as fractures, scoliosis, and osteoarthritis. The CNNs have been trained to accurately classify spinal curves in adolescent idiopathic scoliosis and assist in identifying disc degeneration from lumbar MRI scans. In paediatric fracture detection, multicentre studies have shown that AI-assisted radiography improves clinician sensitivity and diagnostic consistency [1,2].

Several AI systems trained on large datasets of joint radiographs have achieved accuracy levels comparable to, or exceeding, those of expert radiologists. Some studies have reported area under the curve (AUC) values above 0.90 for detecting hip and knee osteoarthritis in specific validation cohorts, while others have shown promising performance in controlled validation settings in detecting fractures of the wrist, shoulder, and hip. These results are not confined to research settings; real-world clinical studies report that AI tools can enhance radiological decision-making in busy orthopaedic departments [8].

Surgical planning and implant identification

Artificial intelligence is increasingly being incorporated into preoperative and intraoperative workflows. Smart navigation systems are now available to assist with implant positioning and soft tissue balancing. These systems improve accuracy in component alignment and reduce variability in surgical execution. Deep learning algorithms have demonstrated high reported accuracy in identifying implant models from radiographs in selected datasets, supporting pre-revision planning and reducing reliance on operative records [3].

Research also suggests that AI-assisted surgical planning tools may reduce the number of intraoperative corrections to templated surgical plans and improve procedural efficiency. Although these tools are still in development, they hold promise for improving surgical outcomes, reducing complications, and personalising implant selection. However, long-term outcome superiority of AI-assisted surgical technologies over conventional techniques has not yet been definitively established.

Biomechanics and gait analysis

The AI-based motion analysis systems are increasingly replacing traditional gait labs in both research and clinical rehabilitation. These systems typically use wearable inertial sensors, smartphone video analysis, or depth cameras combined with machine learning models to extract spatiotemporal gait parameters. This provides an accessible, cost-effective means of capturing functional outcomes in both inpatient and outpatient settings [4]. The AI-driven gait monitoring allows for continuous, real-world rehabilitation tracking, which is particularly valuable in post-operative recovery studies and in patients with neuromuscular conditions. Some systems also provide real-time feedback to clinicians and patients, allowing for individualised gait retraining protocols.

Predictive modelling in clinical research

The use of AI to predict clinical outcomes is expanding rapidly. In the context of osteoporosis and fragility fractures, machine learning algorithms have been developed to estimate fracture risk based on demographic, clinical, and imaging data. Similarly, predictive models are being used to forecast hospital length of stay, complication rates, and even overall treatment costs. Many of these models use algorithms such as support vector machines, random forests, or naive Bayes classifiers trained on large administrative datasets. [9]

While the majority of predictive models remain retrospective in design, their ability to stratify patient risk and assist in preoperative counselling has been recognised. Further validation in prospective, multicentre studies is warranted to confirm these findings and evaluate their real-world applicability. Additionally, model calibration, external validation, and real-world clinical usability remain underreported in many predictive modelling studies.

AI in academic and research workflows

Artificial intelligence is also beginning to transform how orthopaedic research is conducted. Large language models such as ChatGPT are being explored for use in literature summarisation, manuscript drafting, and educational material development. These tools can save time by synthesising existing content, generating initial drafts, and assisting non-native English-speaking authors with writing fluency. However, the use of generative AI raises significant concerns.

However, tools like ChatGPT have underperformed in orthopaedic examination simulations, scoring well below human trainees [10,11]. Accordingly, their use in academic settings requires strict oversight. Most medical journals now mandate full disclosure of AI-assisted writing under COPE and ICMJE guidelines. An additional concern is the emergence of 'AI humanisers', i.e., tools that rewrite machine-generated text to bypass detection by AI-detection systems. While they may improve clarity, their misuse to obscure AI authorship represents a risk to academic integrity [12].

Current limitations and challenges

Despite its promising applications, several limitations continue to restrict the widespread integration of AI in orthopaedics. One of the foremost concerns is the issue of data quality and bias. Many existing AI models are developed using relatively small, institution-specific datasets that lack adequate demographic and clinical diversity. This narrow data representation reduces the external validity of such models and limits their reproducibility when applied across different healthcare settings. [12]

Another major challenge lies in model transparency. A significant proportion of AI algorithms function as 'black box' systems, offering little insight into their decision-making processes. This lack of interpretability poses difficulties for clinicians and researchers who must ultimately justify treatment choices and maintain accountability for patient outcomes. Without greater transparency, widespread clinical trust and acceptance remain difficult to achieve.

Ethical and legal uncertainties also represent significant obstacles. Questions surrounding patient data ownership, liability for AI-assisted clinical decisions, and the adequacy of informed consent in algorithmic analysis are yet to be fully addressed. The absence of robust regulatory and legal frameworks further complicates the safe deployment of these technologies in clinical practice. [13]

In addition, the problem of generalisability continues to limit adoption. Models validated in one specific population often underperform when applied to different patient groups or healthcare systems. This highlights the urgent need for multicentre collaborations, larger datasets, and rigorous external validation protocols before these tools can be confidently implemented in real-world practice. [14]

Finally, the academic use of generative AI presents its own risks. While these tools can expedite scientific writing, their improper use has raised concerns regarding plagiarism, fabrication of data, and diminished authorship accountability. Such misuse undermines the credibility of research and poses ethical challenges for academic publishing [14].

Gaps in the evidence base

While the number of AI-focused orthopaedic studies is steadily increasing, the majority remain retrospective investigations primarily devoted to model development. Although methodological limitations such as limited external validation have been discussed previously, there remains a notable shortage of prospective clinical trials that directly evaluate the impact of AI-assisted decision-making on patient care. Such trials are essential for moving beyond proof-of-concept studies and establishing clinically meaningful benefits.

Equally lacking are studies that prioritise patient-centred outcomes, such as improvements in quality of life, pain reduction, and functional recovery. These measures are fundamental to determining whether AI systems deliver tangible value to patients, yet they are frequently overlooked in current research. Another gap lies in cost-effectiveness analyses. Few studies have rigorously compared the economic implications of AI-based tools against standard care pathways, leaving uncertainty regarding their financial sustainability and long-term feasibility in resource-constrained healthcare systems.

Finally, reports on real-world integration of AI systems into orthopaedic workflows remain limited. The practical challenges of implementation, including training requirements, system interoperability, and clinician acceptance, are seldom addressed in published studies, despite being critical to successful adoption. Few studies have systematically evaluated how clinicians interpret and interact with AI outputs during real-time clinical decision-making, highlighting an important gap in understanding human-AI collaboration in orthopaedic practice. Current orthopaedic AI literature includes studies ranging from proof-of-concept model development to early clinical validation, with relatively few prospective studies evaluating real-world clinical deployment and patient-centred outcomes.

Future research directions

To responsibly advance AI integration into orthopaedic research and practice, several key priorities must be addressed. First, there is a pressing need to strengthen data infrastructure. The development of large, diverse, and ethically sourced datasets will be critical for training and validating robust AI models that are generalisable across populations and healthcare systems.

Equally important is the promotion of explainable AI (XAI) frameworks. By providing interpretable outputs, such systems can enhance transparency, improve clinician trust, and facilitate safe decision-making in clinical settings. Without explainability, even highly accurate models risk rejection due to a lack of accountability.

The implementation of prospective, multicentre clinical trials represents another crucial priority. These studies will enable rigorous evaluation of AI tools in real-world contexts, moving beyond theoretical performance metrics to assess their actual clinical utility and patient outcomes.

Regulatory oversight will also play a central role. The establishment of orthopaedic-specific ethical and legal standards is essential to clarify issues such as liability, informed consent, and patient data protection. Such frameworks will help safeguard both clinicians and patients as AI becomes more deeply embedded in care delivery.

Finally, more research is needed to examine workflow integration. Studies should focus on how AI tools influence clinical efficiency, reduce diagnostic delays, and impact cost-effectiveness. Understanding these practical aspects will determine whether AI can genuinely enhance and not burden orthopaedic practice.

Patient-centred considerations

Artificial intelligence has the potential to improve patient care by enabling earlier diagnoses, reducing human error, and tailoring rehabilitation. Automated scoliosis curve measurement may reduce diagnostic variability, and AI-based fracture detection could lower missed injury rates in paediatric care. Gait analysis platforms offer patients real-time feedback during recovery. However, models trained on non-diverse data may misclassify underrepresented populations, and over-reliance on AI may erode the clinician-patient relationship. Note, as this report represents a narrative synthesis rather than a systematic review, formal risk-of-bias assessment, quantitative pooling of outcomes, and hierarchical grading of evidence were not performed.

## Discussion

Artificial intelligence is beginning to reshape orthopaedics by improving how we diagnose conditions, plan surgeries, and analyse outcomes. While current models show promising results in areas like fracture detection, implant recognition, and gait analysis, most of the evidence still comes from early-stage or retrospective studies. Few tools have been tested in prospective, real-world environments, limiting confidence in their everyday clinical use [1-5]. The transition from technical validation to demonstrable patient-centred benefit remains the central scientific challenge in current orthopaedic AI research. The diverse applications of AI in orthopaedics, ranging from diagnostic imaging to surgical planning and predictive modelling, are summarised in Table 1.

**Table 1:** Overview of AI applications in orthopaedics across major clinical and research domains This conceptual table summarizes key application areas of AI identified through narrative synthesis of PubMed-indexed literature reviewed in this report. The domains correspond to major thematic sections discussed in the manuscript, including diagnostic imaging, surgical planning, predictive clinical modelling, biomechanics and gait analysis, and academic research workflows. Representative applications, advantages, and challenges listed in each column are derived from findings across multiple cited studies and are intended to provide readers with a structured overview of current capabilities, limitations, and future research priorities in orthopaedic AI.

Domain	Representative applications	Key advantages	Current challenges
Imaging and diagnostics	Fracture detection, osteoarthritis grading, tumor classification	Rapid triage, improved diagnostic accuracy, and workflow automation	Dataset bias, limited generalizability, need for external validation
Surgical planning	Robotic-assisted arthroplasty, preoperative templating, and implant tracking	Enhanced precision, reduced intraoperative variability	High implementation cost, lack of long-term outcome data
Predictive modeling	Infection risk stratification, healing trajectory prediction	Supports personalized decision-making, proactive care	Small or homogenous datasets, model overfitting risk
Biomechanics and gait	Wearable-sensor gait monitoring, posture analysis	Affordable motion tracking enables remote rehab	Lack of standard protocols, variability in data quality
Research and education	Literature screening, manuscript generation, and content summarisation	Streamlines writing and review, aids non-native speakers	Risk of fabricated references, unclear authorship roles

**Figure 1:**
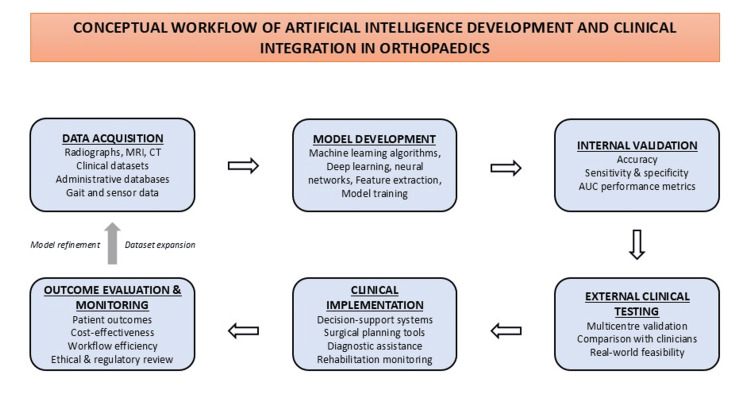
Conceptual workflow of AI development and clinical integration in orthopaedics This figure illustrates the typical development pathway of AI applications in orthopaedics. The process begins with data acquisition from imaging, clinical, administrative, or sensor-based sources, followed by model development using machine learning and deep learning techniques. Subsequent stages include internal validation through performance metrics, external clinical testing in real-world settings, and eventual clinical implementation as decision-support or surgical planning tools. Continuous outcome evaluation and regulatory oversight inform ongoing model refinement and dataset expansion. The workflow emphasises the iterative and validation-dependent nature of artificial intelligence integration into orthopaedic practice. This flowchart is created by the author and is not a reproduction or adaptation from any article, website, or previously published source.

As demonstrated in Table 1, diagnostic imaging currently represents the most mature domain of orthopaedic AI research, with several models demonstrating high diagnostic accuracy and clinical feasibility. In contrast, predictive modelling and biomechanics applications remain promising but less standardised, with ongoing challenges related to validation and data consistency. Academic and research workflow applications represent an emerging area, offering efficiency benefits while simultaneously raising concerns regarding authorship transparency and research integrity. Together, these domains highlight the broad but uneven maturity of AI implementation in orthopaedics. The development and clinical integration pathway of AI applications in orthopaedics is illustrated conceptually in Figure 1.

A key challenge lies in the quality and scope of training data. Many models are built on narrow datasets, which may not reflect broader patient populations. This can introduce bias and reduce model accuracy when applied elsewhere. Additionally, most deep learning systems offer little insight into how decisions are made, a barrier to trust and regulatory approval.

In the academic space, generative AI tools are gaining traction but raise concerns. While useful for drafting and summarising, they can produce inaccurate references or misleading statements. Responsible use, including transparency and human oversight, is essential to maintaining research integrity [9-11]. Looking ahead, AI will likely become a support tool rather than a replacement for clinical judgement. For meaningful integration, future research should focus on diverse data collection, explainable models, and trials that measure impact on patient care. Ethical and legal frameworks will also be key to safe and sustainable adoption.

As AI becomes more embedded in orthopaedic practice, we are not merely adopting a tool; we are reshaping the rhythm of care itself. Artificial intelligence does not tire, does not doubt, and does not forget, yet it also does not feel. A scan read by an algorithm may be fast and precise, but it cannot hear the hesitation in a patient's voice or recognise the unspoken weight of a diagnosis. The challenge ahead is not to teach machines to think like us, but to remember why we think the way we do. In choosing what we hand over to automation, we are also choosing what remains deeply, defiantly human. And perhaps, in that balance, lies the true art of modern medicine.

## Conclusions

Artificial intelligence is increasingly influencing orthopaedic research and clinical practice, although most current applications remain in early developmental or validation stages. It brings speed, precision, and tireless memory to our work, illuminating patterns that might otherwise be overlooked and offering predictions at a scale no human could process alone. Yet, with every tool we gain, it is important to ask not only what it can do but also how it changes the way we practise medicine.

This technology holds immense promise, but it is not a cure-all. Current evidence supporting AI in orthopaedics is largely derived from retrospective or technical validation studies, with limited prospective data demonstrating measurable improvements in patient-centred outcomes. Its insights are only as reliable as the data that trains it, and its effectiveness can be limited by bias, blind spots, or lack of contextual awareness. While current evidence remains preliminary, future advancements in AI may continue to reshape how orthopaedic care is delivered.

As we look to the future, the question is not whether AI will shape orthopaedics, for it already does, but rather how we will shape it in return. The challenge is to ensure that AI has the potential to enhance clinical judgement without replacing it, improve efficiency without eroding compassion, and ultimately strengthen the human foundation of medicine built on presence, empathy, and trust.
